# Superficial Nephrons in BALB/c and C57BL/6 Mice Facilitate *In Vivo* Multiphoton Microscopy of the Kidney

**DOI:** 10.1371/journal.pone.0052499

**Published:** 2013-01-21

**Authors:** Ina Maria Schießl, Sophia Bardehle, Hayo Castrop

**Affiliations:** 1 Institute of Physiology, University of Regensburg, Regensburg, Germany; 2 Institute of Physiology, Department of Physiological Genomics, Ludwig-Maximilians-University, Munich, Germany; 3 Institute for Stem Cell Research, Helmholtz Zentrum, Munich, Germany; Aarhus University, Denmark

## Abstract

Multiphoton microscopy (MPM) offers a unique approach for addressing both the function and structure of an organ in near-real time in the live animal. The method however is limited by the tissue-specific penetration depth of the excitation laser. In the kidney, structures in the range of 100 µm from the surface are accessible for MPM. This limitation of MPM aggravates the investigation of the function of structures located deeper in the renal cortex, like the glomerulus and the juxtaglomerular apparatus. In view of the relevance of gene-targeted mice for investigating the function of these structures, we aimed to identify a mouse strain with a high percentage of superficially located glomeruli. The mean distance of the 30 most superficial glomeruli from the kidney surface was determined in 10 commonly used mouse strains. The mean depth of glomeruli was 118.4±3.4, 123.0±2.7, 133.7±3.0, 132.3±2.6, 141.0±4.0, 145.3±4.3, 148.9±4.2, 151.6±2.7, 167.7±3.9, and 207.8±3.2 µm in kidney sections from 4-week-old C3H/HeN, BALB/cAnN, SJL/J, C57BL/6N, DBA/2N, CD1 (CRI), 129S2/SvPas, CB6F1, FVB/N and NMRI (Han) mice, respectively (n = 5 animals from each strain). The mean distance from the kidney surface of the most superficial glomeruli was significantly lower in the strains C3H/HeN Crl, BALB/cAnN, DBA/2NCrl, and C57BL/6N when compared to a peer group consisting of all the other strains (p<.0001). In 10-week-old mice, the most superficial glomeruli were located deeper in the cortex when compared to 4-week-old animals, with BALB/cAnN and C57BL/6N being the strains with the highest percentage of superficial glomeruli (25% percentile 116.7 and 121.9 µm, respectively). In summary, due to significantly more superficial glomeruli compared to other commonly used strains, BALB/cAnN and C57BL/6N mice appear to be particularly suitable for the investigation of glomerular function using MPM.

## Introduction

The key functional determinates of renal function in vivo, such as renal blood flow, capillary blood flow, glomerular filtration rate, and tubular reabsorption, are usually assessed at the whole organ level. In turn, the structural basis of renal function, such as the structure of the filtration barrier or the tubular system, is classically investigated ex vivo by microscopic evaluation of fixed or frozen tissue. In contrast to conventional approaches, multiphoton microscopy (MPM) is a unique method for addressing both renal function and structure in near-real time in the live animal [1]. However, the use of MPM in kidney research limits imaging of functionally relevant structures located deep within the organ, such as the renal medulla. These structures are beyond the reach of the current excitation MPM lasers for in vivo applications [2]. As a consequence, MPM is limited to the renal cortex, where imaging with reasonable resolution is only possible in the most superficial tissues. The limited range of MPM imaging in the kidney is related to high light scattering and absorption by the heterogeneous renal tissue, and this range is markedly more restricted than in other organs with optically clear parenchyma, such as the brain [3], [4].

Although the aforementioned technical limitation is of minor relevance for imaging superficially located structures such as the proximal tubule, the distal convoluted tubule, and the most superficially located cortical collecting duct, it hinders the investigation of other functionally highly relevant structures, such as the glomerulus and the juxtaglomerular apparatus (JGA). Therefore, MPM imaging of the glomerulus and the JGA has primarily been performed in Munich-Wistar-Froemter (MWF) rats, a rat strain that features many superficially located glomeruli [5], [6]. However, the use of MWF rats in kidney research is problematic, because this rat strain suffers from progressive deterioration of kidney function, due to the development of proteinuria, glomerulosclerosis, and hypertension [5], [6], [7], [8], [9]. In mice, MPM imaging of superficially located glomeruli has been reported in intact BL/6 mouse kidneys, but imaging has been preferentially performed in the kidneys of young animals because the number of glomeruli close to the kidney surface decreases considerably as the organ grows [10]. At present, no systematic data are available in terms of glomerular depth in different mouse strains. Consequently, we aimed to identify a mouse strain that features close-to-surface glomeruli, similar to those observed in MWF rats. Such a mouse strain would be preferable for all MPM evaluations of the glomerulus and would allow back-crossing of relevant transgenic strains into the identified ideal genetic background. For this purpose, 10 commonly used mouse strains were evaluated in terms of the depth of their most superficially located glomeruli. In addition, we determined the influence of growth and age on the distance of glomeruli from the kidney surface.

We found substantial differences in the number and average depth of the most superficially located glomeruli in the various mouse strains. In adult mice, BALB/c and C57BL/6 appear to be the most suitable strains for MPM imaging. However, when imaging the kidneys of young animals, the preferred strains are BALB/c, C3H/N, and DBA/2. Despite marked strain differences, MWF rats exhibited superior imaging conditions than all mouse strains evaluated in the current study.

## Methods

### Animals

Mice 4 and 10 weeks of age (n = 5 from each age group) of the following 10 commonly used strains were analyzed: 129 [129S2/SvPas], BL/6 [C57BL/6N], CB6F1 [CB6F1], C3H/N [C3H/HeN], NMRI [NMRI (Han)], SJL [SJL/J], DBA/2 [DBA/2N], BALB/c [BALB/cAnN], FVB/N [FVB/N] and CD-1 [CD1 (CRI)]. The abbreviated versions of the strain names will be used throughout the manuscript. Both male and female mice were used in this study (Charles River Laboratories International, Inc.). Animal care and experimentation were approved by the local government, *Regierung der Oberpfalz* (54-2532.1-11/12), and carried out in accordance with National Institutes of Health principles as outlined in the *Guide for the Care and Use of Laboratory Animals.* All animal surgery was performed under ketamine/xylazine anesthesia, and anesthetized animals were sacrificed by cervical dislocation at the end of the experiments. Animals were not treated with any drug or intervention before anesthesia.

### Histology

**Figure 1:**
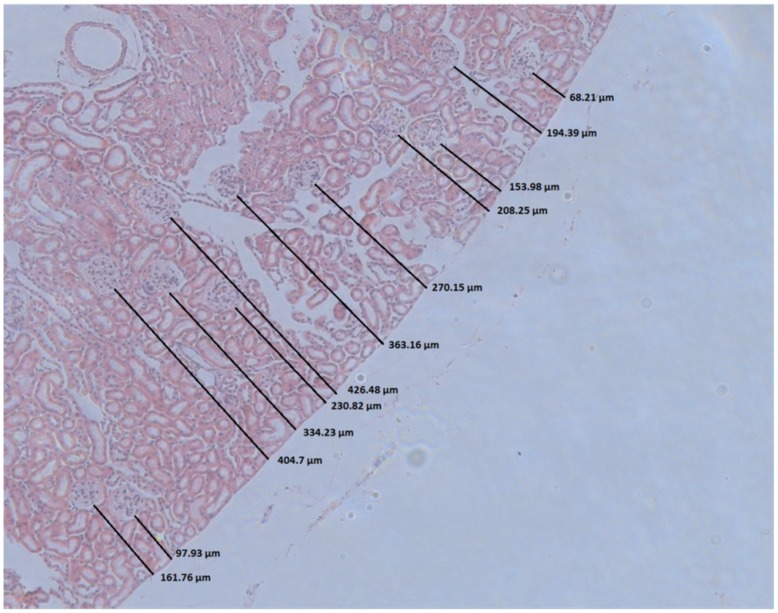
Determination of the depth of glomeruli from the kidney surface of a mouse. The length of the shortest vertical line from the kidney surface to the closest aspect of the glomerulus was determined.

Animals were perfused with 40 ml of 3% paraformaldehyde solution in phosphate buffered saline (pH 7.2) at a constant perfusion pressure of 120 mm Hg. After embedding in paraffin, areas from the middle of the kidney were sliced (6 µm) and stained with hematoxylin and eosin stain (H&E). The depth of all glomeruli within one section of the cortex was measured using Fijii Image J and arranged according to the distance from the organ’s surface. To measure the depth of the glomeruli, the length of the shortest connecting line from the kidney surface to the closest aspect of the glomerulus was determined (fig. 1). The 30 most superficial glomeruli of each section were used for further analysis. The total number of glomeruli analyzed per section was between 89 and 182.

### Multiphoton Microscopy

Animals were anesthetized by i.p. injections of ketamine/xylazine (100/10 mg/kg of body weight). Polyethylene tubing was inserted into the trachea to facilitate breathing, and the jugular vein was cannulated for dye injections. For imaging of the kidney, the left kidney was exposed by a small flank incision. For the liver a horizontal incision right under the sternum was performed and the organ was exteriorized by gently applied pressure on the abdomen. For in vivo live imaging in the adult mouse brain a chronic cranial window was prepared as previously described [18]. After unilateral craniotomy of 3 mm in diameter above the somatosensory cortex, a glas cover slip (5 mm in diameter) was permanently sealed with dental acrylic. The animal was then placed upon an inverse Zeiss LSM 710 NLO confocal fluorescence microscope (Carl Zeiss Jena GmbH, Jena, Germany), which was equipped with an applicable warming plate to maintain the animal’s body temperature at 37°C. Excitation was achieved using a Chameleon Ultra-II MP laser (Coherent Deutschland GmbH, Dieburg, Germany) at 860 nm with a laser power of 3200 mW. Emission was collected using external detectors (nondescanned detectors with filterset 1: beamsplitter 500–550, longpass (LP) 555 and filterset 2: beamsplitter P 565–610 including mirror) or internal (descanned) detectors. For internal detectors the following filters and beamsplitters were used to visualize different colors: a) blue channel: 404–499/main beamsplitter (MBS) 458; b) green channel: 503–590/MBS 760+; and c) red channel: 596–685/filter wheel (FW)1: rear. The pinhole was opened to the maximum. Images were acquired using a 40× long distance (LD) C-Apochromat 40/1.1 water objective. Second harmonic generation microscopy was used (excitation at 860 nm, emission at 430 nm) for visualization of collagen. Vasculature was labeled by Texas Red conjugated with 70 kDa dextran (20 mg/ml stock solution purified by PD-10 Sephadex G-25M columns [GE Healthcare, Buckinghamshire, UK]). The purified solution (50 µl) was injected i.v., and nuclei were stained using Hoechst 33342 (25 µl of a 10 mg/ml solution). Both dyes were purchased from Invitrogen (Life Technologies Corporation). For the generation of z-stacks, images at various depths of the kidney tissue were acquired, beginning from the very top of the organ, where collagen fibers of the kidney capsule were visible. The gain and offset of the detectors were kept constant, while the laser power was adjusted to keep the light intensity of the images slightly below saturation. Twelve-bit 1024×1024 pixel images were obtained using a pixel dwell time of 3.15 µs and a line average of 5. For acquisition of z-stacks without adjustment of the laser power, the laser power was set to 15%, which gave the best light intensity for the first image of the z-stack in kidney, liver and brain. A z-stack to a tissue depth of 100, 40, and 300 µm for kidney, liver, and brain, respectively was built by acquiring one image/µm. For acquisition of z-stacks with adjustment of laser power, images of the kidney capsule and images 0, 30, 50, 60 and 100 µm below the capsule were acquired. For the liver, were imaging conditions appeared to be worse than in the kidney, images in depths of 0, 10, 20, 30 and 40 µm below the collagen fibers were acquired. In the brain tissue layers in depths of 5, 60, 120, 200 and 300 µm below the surface were investigated. To increase the resolution in deeper areas, the laser power was adjusted to the optimum for each image.

### Statistics

Column statistics were performed using Graph Pad Prism 5. A One-Way ANOVA test was conducted to determine differences in glomeruli depth between the strains. In addition, strains with the most superficial glomeruli were compared to a peer group containing all other strains using an unpaired t-test.

## Results

### Assessment of Excitation-laser Tissue Penetrance in Kidney, Liver, and Brain

**Figure 2:**
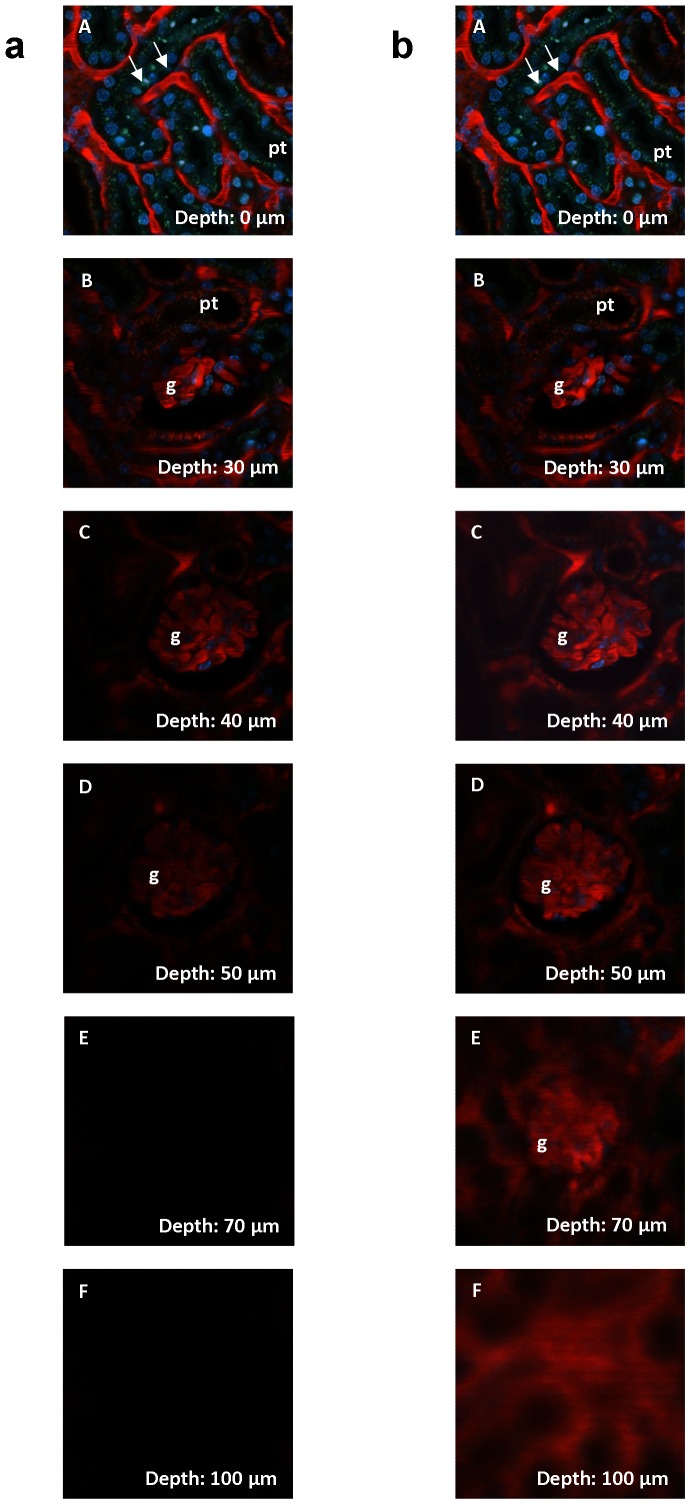
Z-stack through a mouse kidney in vivo, showing typical renal structures like glomeruli (g), proximal tubules (pt) and extraglomerular capillaries (arrows). Images were acquired with internal detectors and gain and offset of the detectors were maintained during the imaging process. (A) – (F) show layers in 0, 30, 40, 50, 70, and 100 µm depth underneath the kidney surface. Capillaries (arrows) are shown in red using Texas Red-labeled 70 kDa dextran. Proximal tubules (pt) showed green autofluorescence. a, left column: Z-stack without adjusted laser power, which was set at 15% of 3.2 W for best imaging conditions of the kidney surface. Without an adjustment of laser power, resolution decreased rapidly so that tissue in a depth of 50 µm was barely accessible. b, right column: Z-stack with adjusted laser power. By increasing the laser power, the limited image quality in deeper layers could be partly compensated. In a depth of 100 µm, maximum laser power produced a bright image, which however was blurry and of low resolution due to light scattering and absorption.

**Figure 3:**
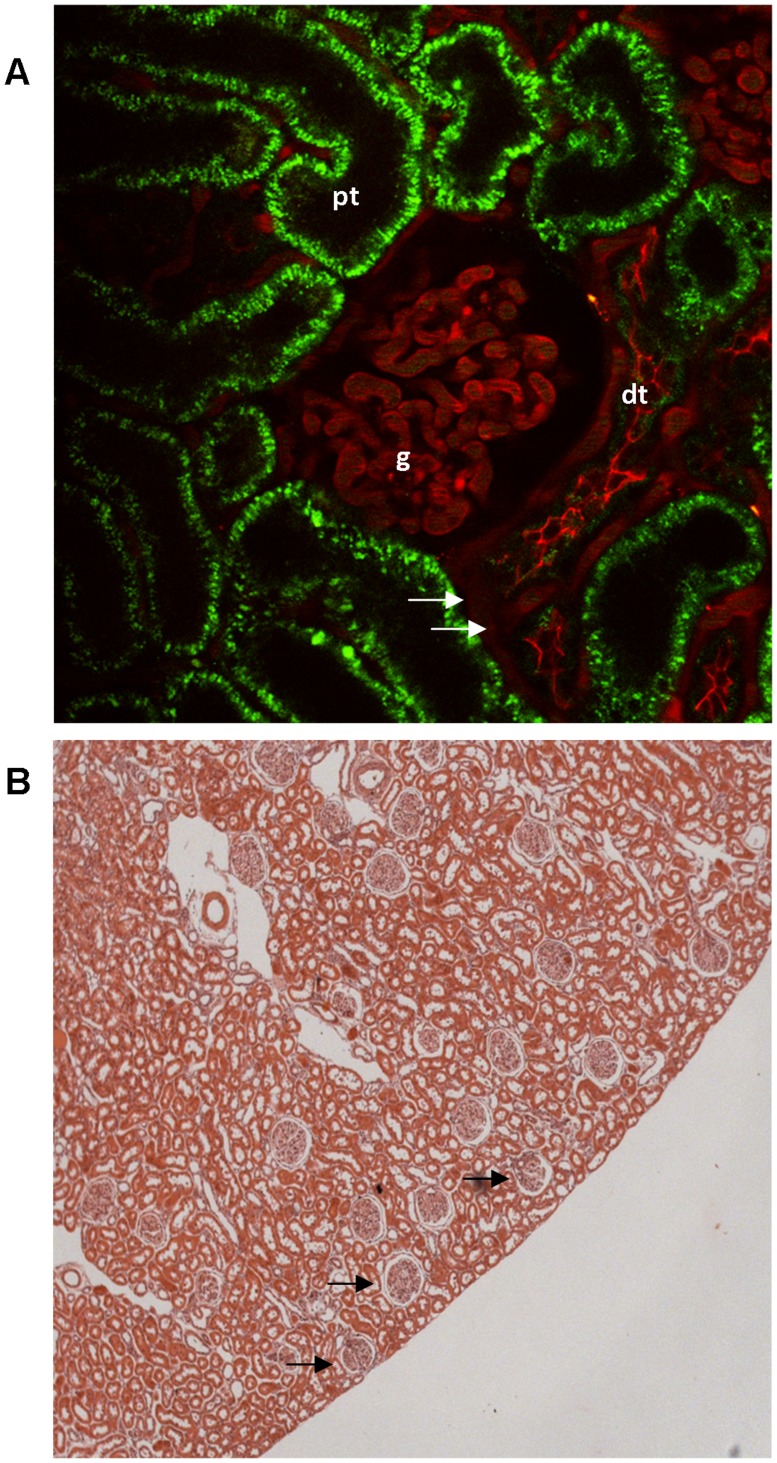
MPM imaging conditions in a kidney of a MWF rat in vivo. (A) Image of a superficial glomerulus (g) and a distal tubule (dt) in the kidney of a MWF rat. Proximal tubules (pt) showed green autofluorescence. Intra- and extraglomerular capillaries (arrows) were visualized with Texas Red-labeled 70 kDa dextran. The glomerulus was located directly underneath the renal capsule allowing high resolution imaging. (B) Histology of a MWF rat kidney section with many superficial glomeruli (arrows).

To assess the imaging conditions of the kidney compared to other organs, we compared in a first experiment the MPM imaging suitability of the kidney, liver, and brain of BL/6 mice under identical imaging conditions, including the same excitation laser, microscope, and acquisition software. MPM imaging was performed in kidneys of BL/6 mice to assess the brightness and image quality as a function of the image’s distance from the kidney’s surface. As shown in fig. 2a, when the laser output power was kept constant, the accessibility of the mouse kidney tissue for MPM imaging decreased rapidly when moving from the kidney surface to deeper layers. Thus, structures located 60 µm from the kidney surface were barely accessible. The image quality, however, could be considerably improved by increasing the laser power for the assessment of structures located deeper in the kidney. By increasing the laser power, structures in the range of 100 µm from the kidney surface became accessible, such as the glomerulus shown in fig. 2b. Although the overall accessibility of mouse and rat kidneys for MPM imaging was similar, the image quality of rat glomeruli at a given depth from the organ’s surface tended to be superior to that of mice (data not shown). In contrast to BL/6 mice, kidneys of MWF rats showed many glomeruli just underneath the renal capsule, allowing high quality imaging of the glomerular structure and of functional features, such as glomerular capillary flow (fig. 3).

**Figure 4:**
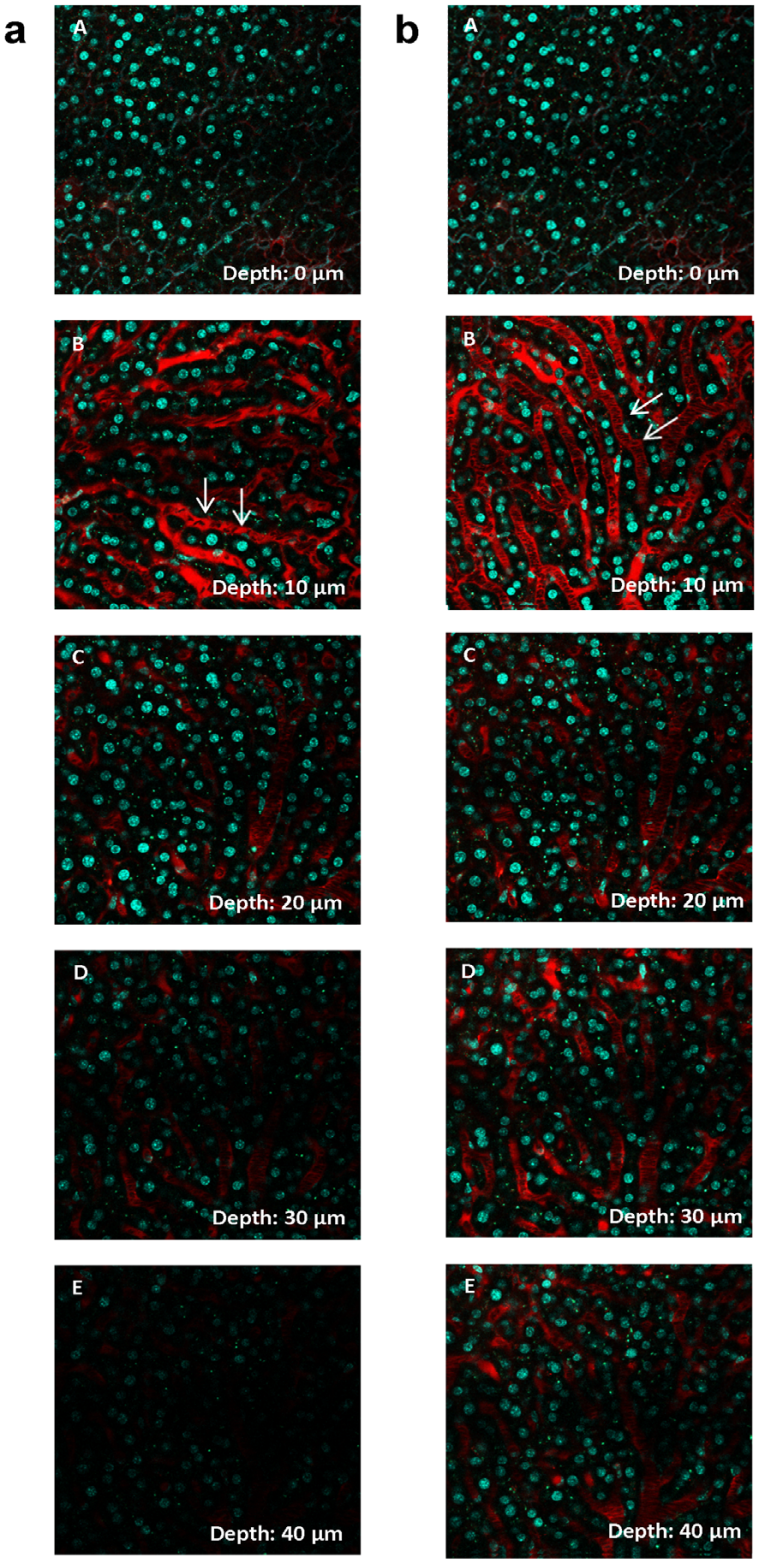
Z-stack through a mouse liver in vivo. Liver images were acquired with internal detectors and gain and offset of the detectors were maintained during the imaging process. (A) – (E) show layers in 0, 10, 20, 30 and 40 µm depth underneath the liver surface. The sinusoids (arrows) were labeled with Texas Red 70 kDa Dextran, the nuclei in cyan by Hoechst 33342. MPM imaging of the liver was highly limited by the light scattering properties of the liver tissue. a, left column: Z-stack without an adjustment of laser power. Image brightness rapidly decreased and imaging was limited to a depth of 40 µm from the organ’s surface. b, right column: Z-stack with adjusted laser power. By increasing the laser output the loss of brightness and resolution can be partly compensated, so that a depth of 40 µm underneath the surface became accessible.

Like for MPM of the kidney, the quality of in vivo MPM imaging of liver structures decreased rapidly with increasing distance from the organ’s surface. For example, a depth of 40 µm was determined as the limit for reliable assessment of vascular blood flow after adjustment of laser output power (fig. 4). Overall, the accessibility of structures located distant from the liver’s surface was markedly more compromised than those in the kidney.

**Figure 5:**
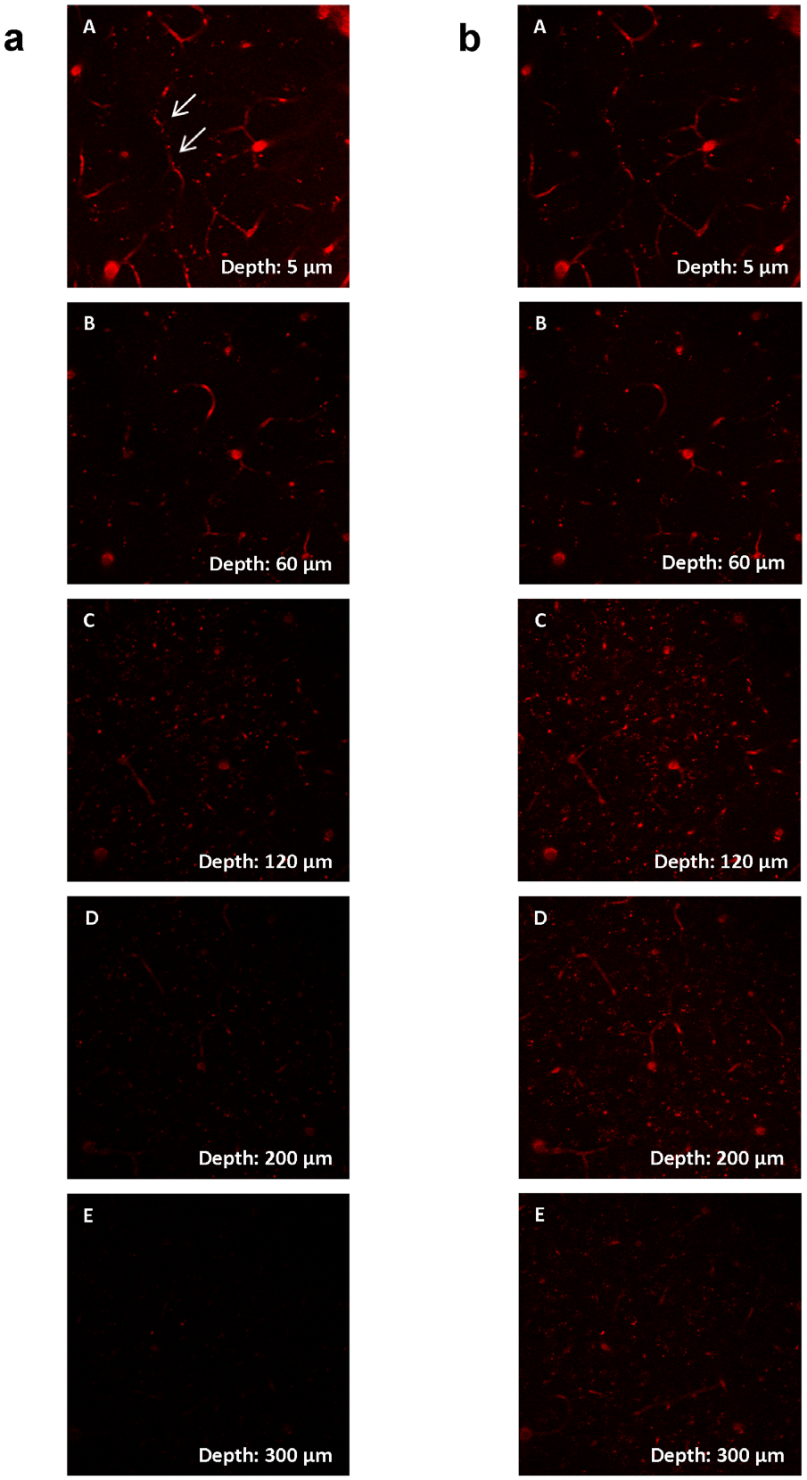
z-stack of the postcentral gyrus of the mouse brain in vivo. (A)–(E) show blood vessels (arrows), stained by Texas Red 70 kDa Dextran in 5, 60, 120, 200 and 300 µm underneath the brain surface. Internal detectors were used with maintained settings for gain and offset. a, left column: Without laser adjustment all blood vessels within the 300 µm range were visible, but appeared darker and blurry below a depth of 200 µm. b, right column: the same region acquired with adjustment of the laser power. In contrast to the liver and kidney a much deeper penetration into the tissue was possible.

In contrast to the kidney and liver, imaging of the mouse brain allowed high quality imaging of structures located as deep as 300 µm from the brain surface (fig. 5). Even depths up to 600 µm were accessible for the brain, as previously shown [3].

### Depth of Glomeruli in 4-week-old Mice of Various Strains

**Figure 6:**
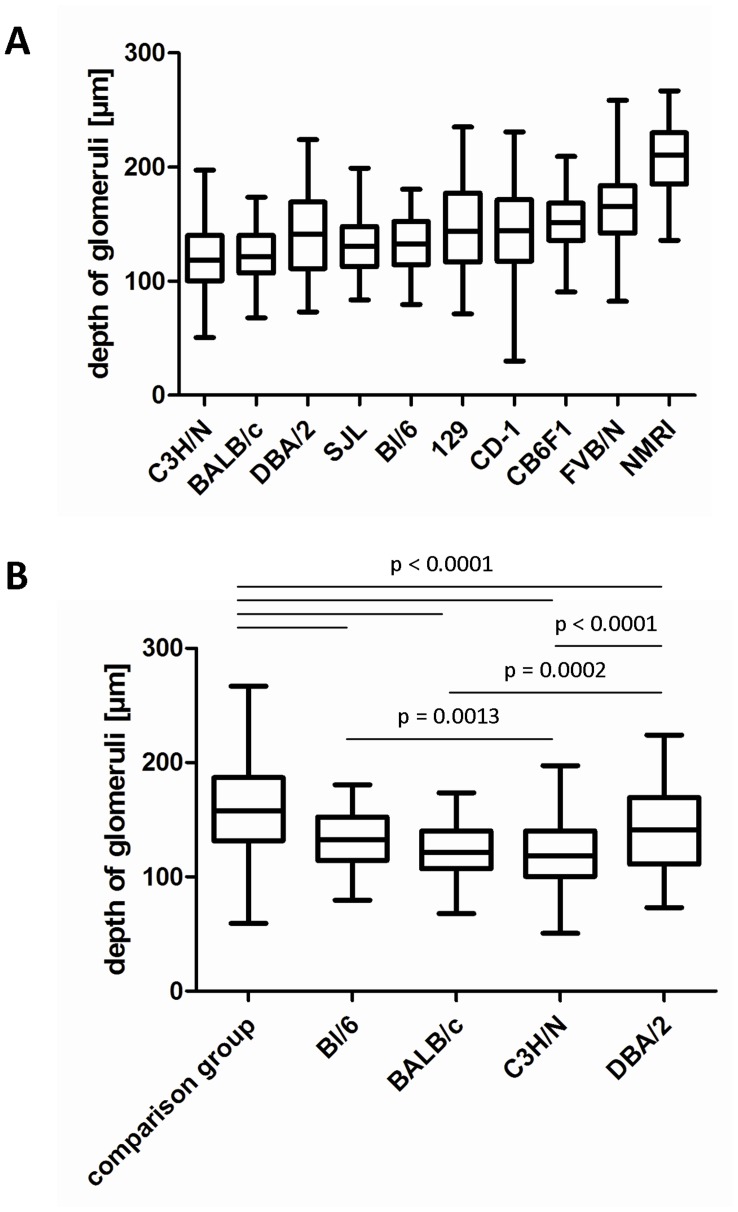
Depth of glomeruli in 4-week-old juvenile mice. Box-plot shows mean±25% (boxes) and the 5–95% percentile (whiskers). (A) Box-plot of all ten strains arranged according to the 25% percentile. (B) Box-plot of the peer group (129, CB6F1, NMRI, SJL, FVB, CD-1) vs. Bl/6, BALB/c, C3H/N and DBA/2.

To identify mouse strains with superficial glomeruli and ideal MPM imaging conditions similar to those observed in MWF rats, 4-week-old juvenile mice of 10 different strains were evaluated in a first set of experiments. Mice of both genders were analyzed as one data set because no gender differences were detected. For MPM, suitable glomeruli are located randomly within the tissue and only the most superficial ones are usually used for imaging. Thus we measured the depth of all glomeruli within one section and only analyzed the depth of the 30 most superficial glomeruli per kidney section. The mean distances from the kidney surface of the 30 most superficial glomeruli were 118.4±3.4, 123.0±2.7, 133.7±3.0, 132.3±2.6, 141.0±4.0, 145.3±4.3, 148.9±4.2, 151.6±2.7, 167.7±3.9, and 207.8±3.2 µm for the C3H/N, BALB/c, SJL, BL/6, DBA/2, CD-1, 129, CB6F1, FVB/N, and NMRI strains, respectively (n = 5 animals from each strain) (fig. 6A). In parallel with the mean distance of glomeruli from the kidney surface, the 25th percentile of the most superficial glomeruli differed markedly between the strains. The most superficial glomeruli were observed in a depth of less than 100.4 and 107.0 µm in C3H/N and BALB/c mice, respectively. The deepest glomeruli occurred in FVB/N and NMRI mice, in which the 25% most superficial glomeru1i were located 142.1 and 185.1 µm, respectively, from the kidney’s surface. In summary, the average depth of the most superficial glomeruli was significantly lower in the strains C3H/N, BALB/c, DBA/2, and BL/6 when compared to a peer group consisting of all the other strains (p<.0001; fig. 6B).

### Depth of Glomeruli in 10-week-old Mice of Various Strains

**Figure 7:**
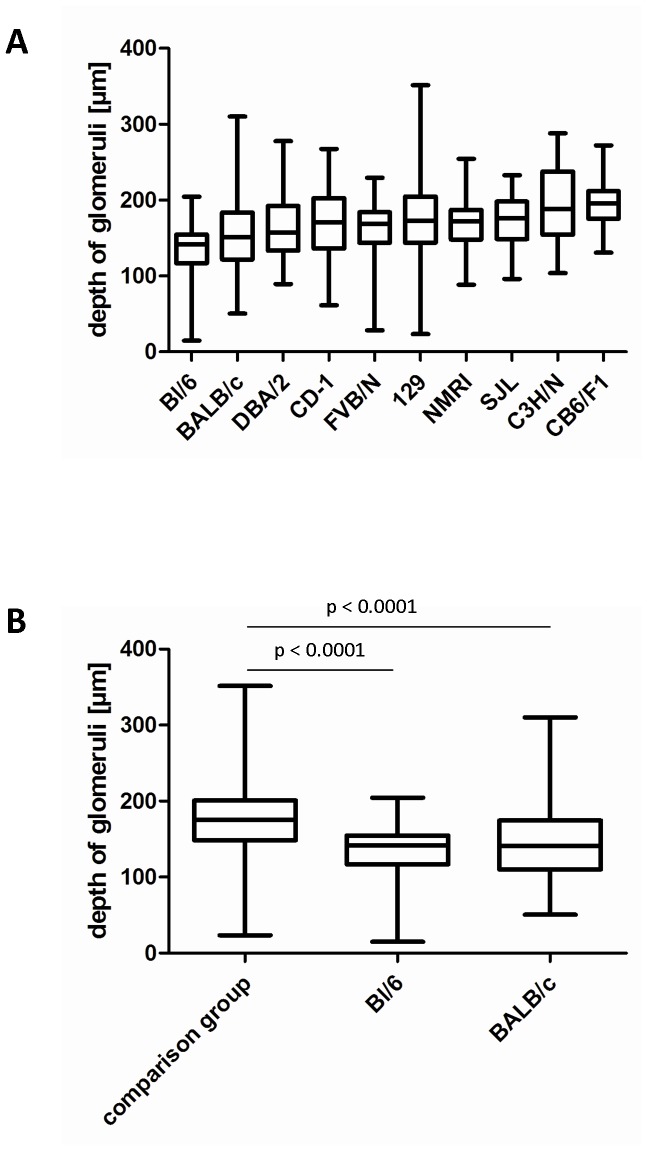
Depth of glomeruli in 10-week-old adult mice. Box-plot shows mean±25% (boxes) and the 5–95% percentile (whiskers). (A) Box-plot of all ten strains arranged according to the 25% percentile. (B) Box-plot of the peer group (129, CB6F1, C3H/N, NMRI, SJL, DBA/2, FVB, CD-1) vs. Bl/6 and BALB/c.

The same analysis of glomerular depth was repeated in adult mice 10 weeks of age. The average kidney weight of all strains was 161.2±2.1 mg in adult 10-week-old mice compared to 112.9±3.8 mg in 4-week-old juvenile animals. As a consequence of kidney growth, the average depth of the 30 most superficial glomeruli in each kidney section increased from 147.0±4.2 µm in 4-week-old mice to 170.1±5.4 µm in 10-week-old mice (p<.001). In adult mice, the average distances from the kidney surface of the 30 most superficial glomeruli were 136.5±3.8, 151.9±3.1, 162.0±3.4, 164.8±4.1, 170.0±4.8, 171.0±3.4, 171.8±3.6, 182.3±5.8, 193.0±5.2, and 197.3±3.4 µm for BL/6, BALB/c, FVB/N, DBA/2, CD-1, SJL, NMRI, 129, C3H/N, and CB6F1 strains, respectively (fig. 7A). The mean distance of the glomeruli from the kidney surface was generally paralleled by the number representing the 25% most superficially located glomeruli, which ranged from 116.7 µm in BL/6 to 175.4 µm in CB6F1 mice (fig. 7A).

Although the 25th percentile of glomeruli depth from the kidney surface was in the borderline range (for BL/6 and BALB/c strains) or out of range (for all other strains) of in vivo MPM, we found single glomeruli that were as close as 15 and 50 µm from the renal surface in the BL/6 and BALB/c strains, respectively. In summary, adult mice from the BL/6 and BALB/c strains showed significantly more superficially located glomeruli when compared to the average of all other strains (p<.0001; fig.7B).

## Discussion

The conditions for in vivo MPM differ in various tissues depending on their optical density and heterogeneity. These tissue-specific conditions limit the penetration depth of the excitation laser at a given wavelength and impact the image quality due to various light scattering phenomena. Although deeper structures can be made accessible by increasing the output power of the excitation laser, the quality of the images is compromised by increased light scattering, which leads to the capitation of bright pictures with low resolution. Furthermore, high excitation laser power results in local phototoxicity, which may compromise the functional features of interest. In this study for the first time we performed a direct comparison for the MPM accessibility of different organs using the same microscope, excitation laser, software and imaging settings. In accordance with previous studies, our comparison of imaging conditions for mouse brain, liver, and kidney tissue revealed that the brain offers excellent imaging conditions compared to other organs, allowing imaging of structures 300 µm and deeper below the pial surface [4], [11]. MPM imaging of the liver, in comparison, was markedly more difficult because structures deeper than 40 µm were barely accessible. This difficulty may be related to specific anatomic features of the liver, such as dense capillaries and sinusoids. In terms of accessibility for MPM the kidney was between the brain and liver. Structures in the kidneys located up to 100 µm from the organ’s surface were accessible. However, depending on the setup, the quality of the pictures in terms of brightness and resolution decreased substantially as a function of the depth of the section. Similar like in most MPM studies of living tissues, we used a water immersion objective. This provides the best optical beam pathway conditions for the in vivo approach, because, first, the kidney was embedded into a 0.9% saline solution for the imaging session and, secondly, the kidney tissue consists mostly of water. Therefore the refraction index of the kidney is more similar to saline than for example glycerol. By using a water immersion objective we minimized the discrepancy between the optical and geometrical depth. All depth measurements in this study are based on the mechanical movement of the stage along the z-axis, which we defined as the penetration depth. It should be noted that in all our MPM imaging, the penetration depth was lower than the free working distance of the applied objective.

The imaging range that we observed for the kidney has also been reported by other groups and might be problematic if assessment of the structure and function of the glomerulus and JGA is desired [2]. As a consequence, most in vivo MPM studies of the glomerulus have been conducted with MWF rats [10], [12], [13], [14], which are characterized by a high concentration of glomeruli just underneath the renal capsule. MWF rats therefore offer ideal conditions for MPM imaging of the glomerulus and JGA. Before MPM became available, MWF rats were traditionally used for micropuncture studies [15], [16], and their renal function has been well documented. One drawback, however, is that MWF rats not only have a desirable high percentage of superficially located glomeruli, they also show a reduced nephron number when compared to other commonly used strains such as Sprague Dawley or Wistar rats [17]. As a model of low nephron number, ageing MWF rats develop hypertension, progressive glomerulosclerosis, proteinuria, and, eventually, deterioration of renal function [6], [7], [17]. For reasons that are not entirely clear, the renal phenotype of MWF rats is more pronounced in male than female rats, making females more suitable for use in studies assessing the structure and function of the kidney and, more specifically, of the glomerulus [8]. These issues could be in part circumvented by the use of the closely related MW Simonsen (MWS) strain. In contrast to MWF rats, MWS rats are less proteinuric and they rarely develop glomerulosclerosis. However, the number of superficial glomeruli in MWS rats is more limited compared to MWF rats [13].

In this study, our aim was to identify a mouse strain with favorable MPM imaging conditions of the kidney similar to those observed in MWF rats. Although imaging of the function and structure of the glomerulus has been done in mice, there are no data available regarding the most suitable strain in terms of superficially located glomeruli. Such a mouse strain may circumvent problems related to compromised kidney function in MWF rats and improve MPM imaging of the large pool of genetically altered mouse models by respective back-crossing into a favorable genetic background. Only few MPM studies of the kidney have been done in mice due to the limited accessibility of the glomeruli. Unilateral ureter obstruction was used in some experiments to move glomeruli to the kidney surface, however this apparently is associated with alteration in glomerular capillary blood flow and generalized inflammation of the kidney [18], [19].

Ten commonly used mouse strains were included in the study, which differed markedly in the number of their most-superficial glomeruli. In general, juvenile mice (4 weeks of age) of all strains showed more superficial glomeruli compared to adult mice (10 weeks of age) and, consequently, appeared more suitable for use in MPM imaging. In this context, it should be noted that most studies using MPM of the mouse kidney were conducted with young animals [10]. The specific use of juvenile animals, however, imposes some critical drawbacks. First, it remains unclear if data on glomerular function obtained from juvenile animals are fully transferable to adult mice. Secondly, functional and structural alterations of glomerular capillaries, podocytes, and Bowman’s capsule in chronic disease models usually become apparent only in the aging kidney. Thirdly, pharmacologic manipulation of glomerular function often requires drug administration over a given time period, which may be too lengthy for young animals. Despite these general limitations, we identified a significantly greater number of superficial glomeruli in the kidneys of young C3H/N, BALB/c, DBA/2, and BL/6 mice in comparison to all other strains, leaving them particularly suitable for MPM imaging of the kidney. The FVB and 129 strains are commonly used for the generation of transgenic mice by pronuclear injection and transgene transfection of embryonic stem cells for the generation of knockout models. These strains ranked in the middle to upper end compared to the other strains and displayed rather few superficial glomeruli. Therefore these standard backgrounds of genetically modified mice are not particularly suited for in vivo MPM of the glomerulus, and backcrossing to a BL/6 or BALB/c strain would be recommended if animals are intended for MPM imaging.

In adult mice (10 weeks of age) the strains BALB/c and BL/6 were preferable because they showed the highest percentage of superficial glomeruli within the range of good MPM imaging conditions. For all mouse strains, the distance of glomeruli from the kidney surface increased in parallel to kidney growth. Additionally, the width of the *cortex corticis*, the area of the cortex underneath the renal capsule that is free of glomeruli, expanded as the kidneys grew. Interestingly, there were striking differences in the relative broadening of the glomeruli-free area during growth between different strains. For example, we found a high number of superficially located glomeruli in the kidneys of 4-week-old C3H/N mice, but this particular strain showed few superficial glomeruli at 10 weeks of age. Thus, the overall suitability of adult C3H/N mice for MPM imaging of renal glomeruli was poor. These apparent differences in the growth of various part of the nephron that leads to the broadening of the subcapsular cortex, which is virtually free of glomeruli, appears to be both strain- and species-dependent. In contrast to some of the mouse strains used in this study, the distribution pattern of glomeruli in the renal cortex in humans and dogs was shown to be largely constant during postnatal growth of the kidney [20], [21].

Although none of the evaluated mouse strains exhibited ideal conditions for MPM imaging similar to those of MWF rats, it should be noted that there was a substantial scatter of glomerular depth in mice. Even if the average depth of the 30 most superficial glomeruli per section was out of reach for MPM, we found scattered superficial glomeruli in many of the strains, with a particular high incidence in BALB/c and BL/6 mice. Given that a mouse kidney has somewhere in the range of 8.000–12.000 glomeruli [22], 5% of the most superficial glomeruli would be estimated at 200. With a surface area of approximately 150 mm^2^, it should be possible to find at least one glomerulus within one mm^2^ that can be easily accessed by MPM with optimal imaging quality.

In summary, the accessibility of renal glomeruli for MPM imaging in mice is highly strain- and age-dependent. Taking together both age groups of this study, the highest numbers of superficial glomeruli readily accessible for MPM were found in BALB/C and BL/6 mice. These strains are preferable for MPM imaging experiments of the kidney, particularly for those studies focused on structural and functional features of the glomerulus.
